# Supplementary material for: Sensitive Quantitative In Vivo Assay for Evaluating the Effects of Biomolecules on Hair Growth and Coloring Using Direct Microinjections into Mouse Whisker Follicles
Source: Biomolecules. 2023 Jul 5;13(7):1076. doi: 10.3390/biom13071076 (PMC10377598; doi:10.3390/biom13071076)
Supplement: Supplementary file 1 [file biomolecules-13-01076-s001.zip › biomolecules-2392389-supplementary.doc.pdf]

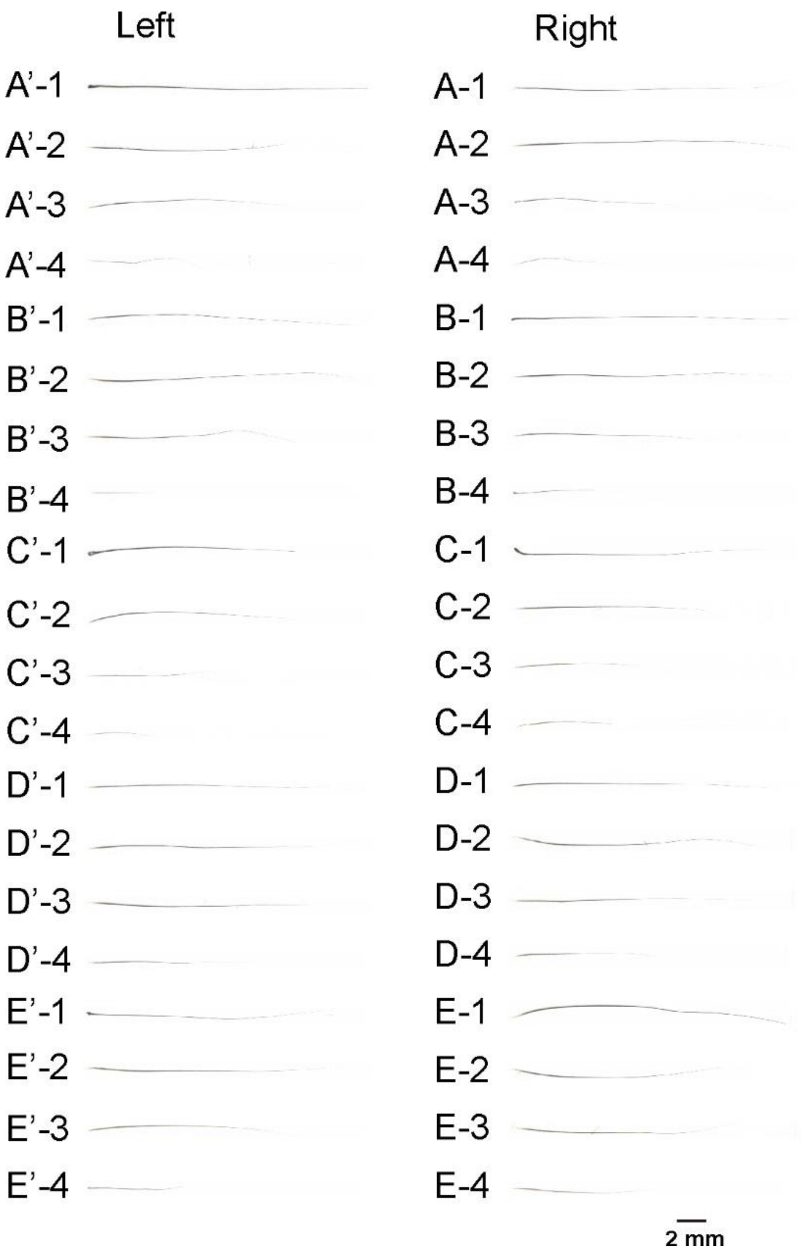

Figure S1. Mouse whiskers are left-right symmetric. All paired whiskers from both the left and right sides of adult male mice were plucked and imaged. If two whiskers grew from the same follicle, only the longer whisker was counted. Scale bars, 2 mm.

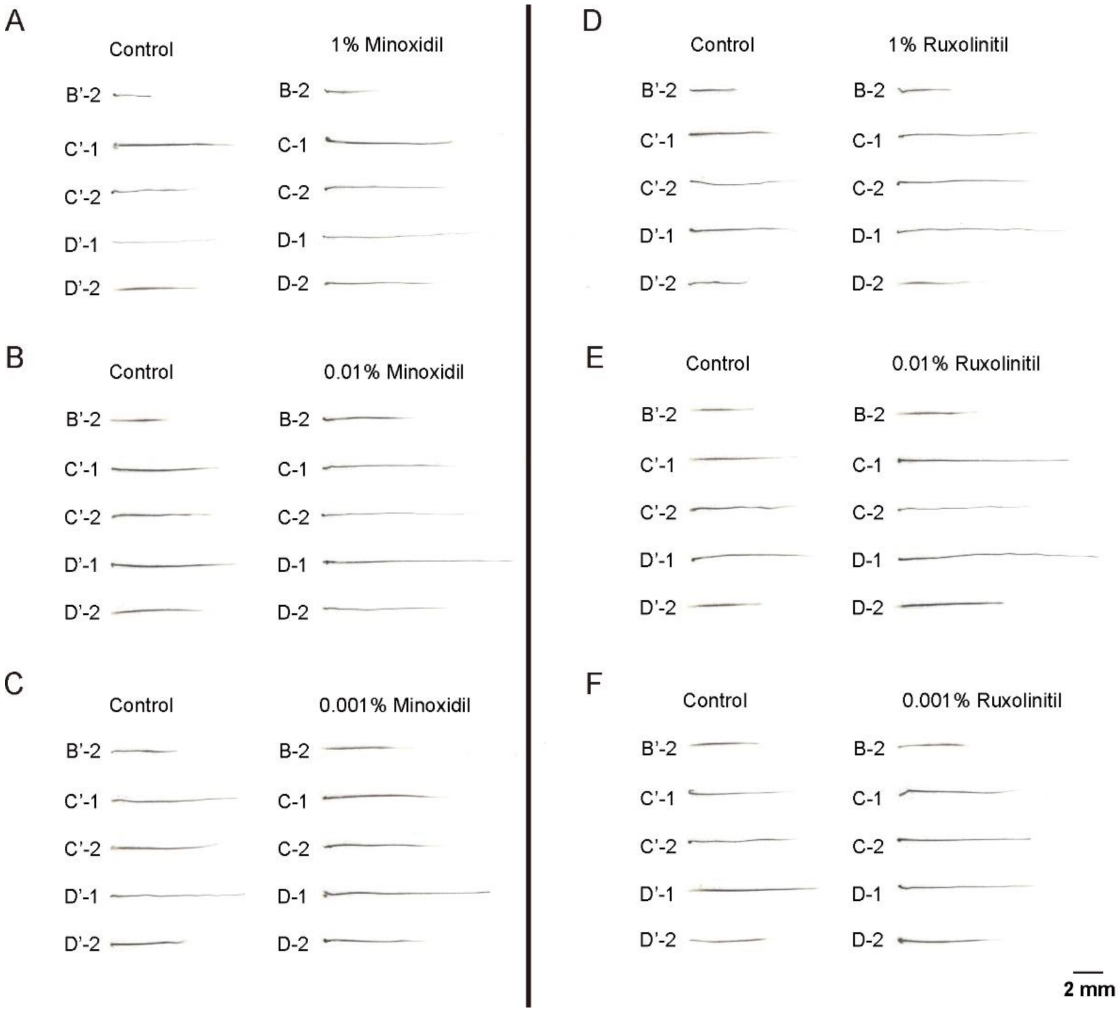

Figure S2. Images of all paired whiskers treated with minoxidil or ruxolitinib. (A- C) Five pairs of large whiskers from adult male mice were plucked and 5 μl of different concentrations of minoxidil (A, 0.001%; B, 0.01%; C, 1%) or a control solvent was injected via micro-syringe into the follicles on the left and right sides on days 1, 3 and 5, respectively. After 11 days, the regrown whiskers were plucked and imaged. (D-F) Like in (A-C), five pairs of regrown whiskers treated with 0.001% (D), 0.01% (E) and 1% (F) ruxolitinib were plucked and imaged. Scale bar, 2 mm.

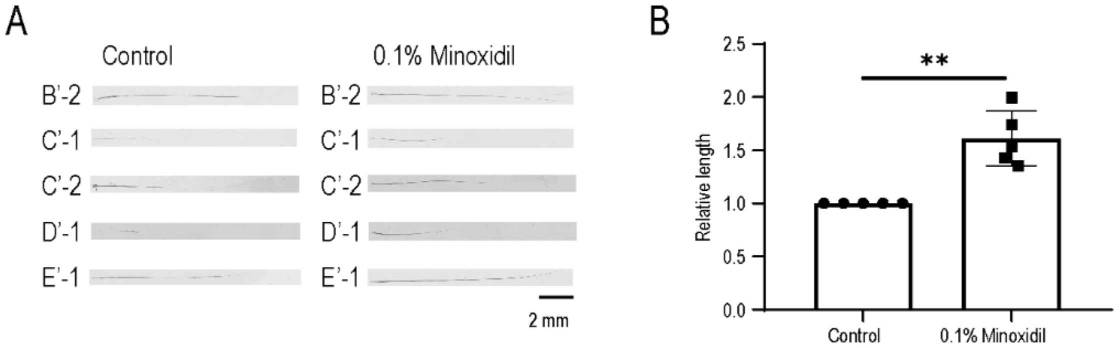

Figure S3. The microinjection of chemicals minoxidil into rat whisker follicle effectively promotes hair growth. (A) Five pairs of large whiskers from adult male rats were plucked and 5 μl of 0.1% minoxidil or a control solvent was injected via micro-syringe into the follicles on the left and right sides on day 0, 3 and 5, respectively. On day 15, the regrown whiskers were plucked and imaged. (B) The length of each regrown whisker was measured, and the statistical difference was evaluated using a two-tailed paired Student's t-test. ** $p < 0.01$. Scale bar, 2 mm.
